# Xanthogranulomatous Appendicitis: A Rare Pathological Finding Associated With Delayed Appendectomy

**DOI:** 10.7759/cureus.68322

**Published:** 2024-08-31

**Authors:** Clark Pitcher, Jee-Hye Choi, John Paulsen, Sergei Dolgopolov

**Affiliations:** 1 Department of Surgery, Icahn School of Medicine at Mount Sinai, New York City, USA; 2 Department of Pathology, Icahn School of Medicine at Mount Sinai, New York City, USA

**Keywords:** interval appendectomy, appendectomy, acute appendicitis, xanthogranulomatous appendicitis, xanthogranulomatous inflammation

## Abstract

Xanthogranulomatous inflammation of the appendix is a rare pathological finding associated with appendicitis and chronic inflammation. Its clinical significance is not fully understood, and diagnosis is primarily based on the histopathological review as imaging findings with CT and ultrasound are non-specific. Here, we present a case of a 64-year-old female with recurrent appendicitis who underwent an appendectomy with final pathological findings consistent with xanthogranulomatous appendicitis (XGA). We discuss the higher reported incidence of XGA in interval appendectomy specimens compared to emergency appendectomies, and how this relates to its proposed pathophysiology. We found that XGA is associated with a more challenging operative field and the need to convert from a laparoscopic to an open procedure, increasing the potential risks of surgical complications. The potential development of XGA should be considered when planning an interval appendectomy as it may impact operative planning, although there is no clear consensus on its clinical significance.

## Introduction

Appendicitis is characterized by inflammation of the vermiform appendix and is primarily caused by obstruction of the appendiceal lumen. Obstruction can be secondary to fecaliths, lymphoid hyperplasia, parasites or tumors, among other etiologies. Inflammation of the wall of the appendix can lead to ischemia, necrosis and perforation, potentially causing the development of a local abscess or generalized peritonitis. Acute appendicitis is one of the most common indications for emergency abdominal surgery, although some patients can be treated with antibiotics and an interval appendectomy [1]. In this report, we present the case of a patient with recurrent appendicitis with final pathological findings after appendectomy consistent with xanthogranulomatous appendicitis (XGA). Xanthogranulomatous inflammation is an uncommon type of chronic inflammation characterized histologically by foamy lipid-laden macrophages among other inflammatory cells [2]. It has been described primarily in the kidney and gallbladder but has been found in various organ systems including the stomach, colon, pancreas, spleen, and genital tracts [3-5]. Xanthogranulomatous inflammation of the appendix is a rare pathological finding with less than 40 cases described in a recent literature review [6]. This case provides an opportunity to explore the clinical significance of XGA and how suspicion for it should increase in patients with recurrent appendicitis as it may lead to a more challenging operative field.

## Case presentation

A 64-year-old female presented with recurrent appendicitis; she also had extensive comorbidities including obesity (body mass index of 30), hypertension, diabetes mellitus, heart failure, a history of pulmonary embolism on anticoagulation therapy, asthma, chronic obstructive pulmonary disease (on long-term home oxygen), spinal stenosis, and alcohol use disorder complicated by pancreatic insufficiency. Her past abdominal surgeries included a hysterectomy and salpingectomy done six years ago.

**Figure 1:**
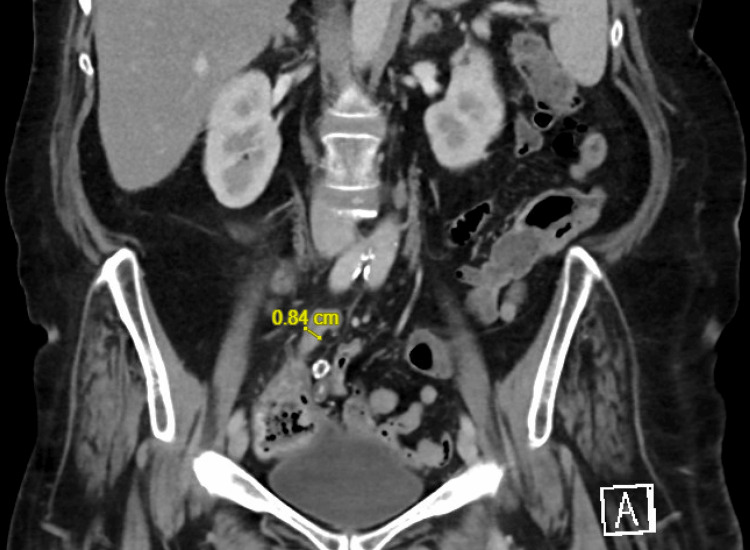
A CT scan showing acute appendicitis with the appendix diameter measuring 0.84 cm and surrounding mild inflammatory changes at the tip

The patient initially presented with perforated appendicitis that was diagnosed with a CT scan and was managed non-operatively with intrabdominal drainage and antibiotics. She was recommended for an interval appendectomy but was lost to follow-up. She then presented again 1.5 years later with lower abdominal pain, nausea, vomiting and imaging findings consistent with appendicitis. The patient was again treated non-operatively with antibiotics, as she declined surgery and was deemed a poor operative candidate given her numerous comorbidities. However, one month later, she presented to the emergency department again with diffuse lower abdominal pain, diarrhea, nausea, and vomiting. She was tachycardic without leukocytosis; a CT scan showed the appendix diameter measuring 0.84 cm with an interval decrease in the mild inflammatory changes at the tip when compared to the study from the prior hospitalization (Figure 1). The clinical picture was consistent with recurrent appendicitis. Given that she had failed medical management and was lost to follow-up in the past, a joint decision between the patient and physician was made to proceed with a laparoscopic appendectomy after holding anticoagulation therapy for 48 hours. A written consent was obtained from the patient.

**Figure 2:**
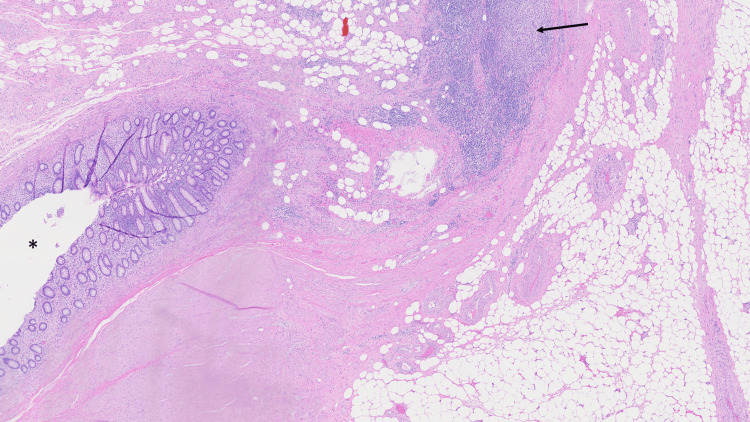
Appendectomy pathology The interval appendectomy specimen showing intramural xanthogranulomatous inflammation (arrow), with the asterisk denoting the appendiceal lumen (hematoxylin & eosin, 20x magnification).

**Figure 3:**
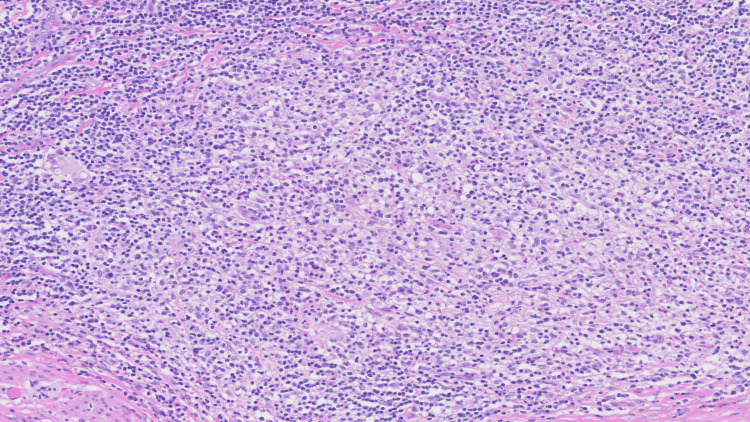
Xanthogranulomatous pathology Xanthogranulomatous inflammation comprised sheets of foamy histiocytes in a background of active chronic inflammation (hematoxylin & eosin, 200x magnification).

The patient was taken to the operating room, where the appendix was identified to be severely inflamed with extremely thick adhesions and phlegmon around the surrounding tissue including the cecum. Further laparoscopic dissection was deemed unsafe with concern for an inadvertent injury, and the procedure was converted to an open appendectomy with an oblique incision made in the right lower quadrant. After open conversion, the appendix was successfully isolated and ligated with a polydioxanone (PDS) Endoloop. Her postoperative course was complicated by ileus and intermittent asymptomatic hypotension, but she was eventually discharged in a stable condition. The pathology report of the appendix showed appendiceal diverticular disease with the background appendix showing mural fibrosis, hemosiderin deposition, focal transmural chronic inflammation and focal intramural xanthogranulomatous inflammation, consistent with xanthogranulomatous interval appendicitis (Figure 2 and Figure 3).

## Discussion

Xanthogranulomatous inflammation of the appendix is a benign condition associated with chronic or recurrent inflammation. While there is no consensus about its pathophysiology, authors have suggested that it may be associated with inflammation, hemorrhage, necrosis, obstruction, and local tissue hypoxia [2,7]. A definitive diagnosis of XGA is based primarily on the histopathological review, as imaging findings with CT and ultrasound are non-specific, and unique features have not been identified yet with XGA, as have been observed in other conditions like xanthogranulomatous cholecystitis (XGC) [8].

XGA should be distinguished from granulomatous appendicitis (GA) that is typically associated with infection (yersinia, tuberculosis, parasites, and fungus, among others), foreign body reaction, Crohn’s disease, sarcoidosis, and interval appendectomy [9]. With the appropriate clinical history, additional studies or workup should be considered to guide further management when GA is discovered on a pathological review. In contrast, XGA is a separate pathological finding with a higher reported incidence in interval appendectomy specimens compared to emergency appendectomies [7]. A recent literature review of cases of XGA supported this, finding that 16 of the 30 patients had an interval appendectomy whereas the rest underwent either urgent or semi-urgent surgery [6]. Another recent study compared the histology slides from emergent appendectomies and interval appendectomies and again found that the specimens from interval appendectomies were more likely to harbor xanthogranulomatous inflammation [10]. No clear difference has been found in the incidence based on sex or age [6].

The gold standard treatment for acute appendicitis is laparoscopic appendectomy. However, the initial non-operative management with antibiotics and a delayed appendectomy can be considered in select patients after a shared decision-making process. A recent clinical trial found that antibiotics were noninferior to appendectomy in cases of uncomplicated appendicitis, but patients with an appendicolith were at an increased risk of complications and appendectomy [11]. The association of XGA with an interval appendectomy brings into question whether XGA is a histological sequela of chronic or recurrent inflammation rather than a separate diagnostic entity, especially since XGA is not associated with any specific preoperative findings. Perhaps the non-operative management of appendicitis increases the risk of development of XGA.

Regardless, XGA has been linked to more challenging operations, as in our case where thick adhesions and a phlegmon surrounding the appendix required conversion to an open appendectomy. Other reports describe similar intraabdominal findings of adhesions, phlegmon, induration, perforation, and abscesses surrounding the appendix, some of which also required a more invasive approach to the surgery [12-14]. Interestingly, a review of cases of XGC found that 11% (67/629) were converted from a laparoscopic to an open approach and over half of all cases required an open surgery [15]. There is a presumed increased risk of complications in cases of XGA since open appendectomies are associated with increased incidences of wound infection and longer hospital stays [16]. Additionally, appendicitis with perforation or abscess changes the surgical wound classification from ‘contaminated’ to the ‘dirty’ category, which has a higher rate of surgical site infections [17].

## Conclusions

In this case, appendectomy was performed semi-urgently, given the recurrent presentation of appendicitis and failure of medical management. It is unknown whether the pathology would have still shown xanthogranulomatous inflammation and whether the patient would have required an open operation if an appendectomy had been performed sooner. However, the potential development of XGA should be considered when planning an interval appendectomy as chronic inflammation may increase the patient’s risk for surgical complications, although further studies are required to elucidate its clinical significance.
